# *In vitro* evaluation of iron oxide nanoparticle-induced thromboinflammatory response using a combined human whole blood and endothelial cell model

**DOI:** 10.3389/fimmu.2023.1101387

**Published:** 2023-04-04

**Authors:** Alexandra Gerogianni, Melissa Bal, Camilla Mohlin, Trent M. Woodruff, John D. Lambris, Tom E. Mollnes, Dick J. Sjöström, Per H. Nilsson

**Affiliations:** ^1^ Linnaeus Centre for Biomaterials Chemistry, Linnaeus University, Kalmar, Sweden; ^2^ Department of Chemistry and Biomedicine, Linnaeus University, Kalmar, Sweden; ^3^ School of Biomedical Sciences, Faculty of Medicine, The University of Queensland, Brisbane, QLD, Australia; ^4^ Department of Pathology and Laboratory Medicine, Perelman School of Medicine, University of Pennsylvania, Philadelphia, PA, United States; ^5^ Department of Immunology, Oslo University Hospital and University of Oslo, Oslo, Norway; ^6^ Centre of Molecular Inflammation Research, Department of Clinical and Molecular Medicine, Norwegian University of Science and Technology, Trondheim, Norway; ^7^ Research Laboratory, Nordland Hospital, Bodo, Norway

**Keywords:** thromboinflammation, iron oxide nanoparticles, endothelial cells, complement activation, complement inhibition, whole blood

## Abstract

Iron oxide nanoparticles (IONPs) are widely used in diagnostic and therapeutic settings. Upon systemic administration, however, they are rapidly recognized by components of innate immunity, which limit their therapeutic capacity and can potentially lead to adverse side effects. IONPs were previously found to induce the inflammatory response in human whole blood, including activation of the complement system and increased secretion of cytokines. Here, we investigated the thromboinflammatory response of 10-30 nm IONPs in lepirudin anticoagulated whole blood in interplay with endothelial cells and evaluated the therapeutic effect of applying complement inhibitors to limit adverse effects related to thromboinflammation. We found that IONPs induced complement activation, primarily at the C3-level, in whole blood incubated for up to four hours at 37°C with and without human microvascular endothelial cells. Furthermore, IONPs mediated a strong thromboinflammatory response, as seen by the significantly increased release of 21 of the 27 analyzed cytokines (p<0.05). IONPs also significantly increased cell-activation markers of endothelial cells [ICAM-1 (p<0.0001), P/E-selectin (p<0.05)], monocytes, and granulocytes [CD11b (p<0.001)], and platelets [CD62P (p<0.05), CD63 (p<0.05), NAP-2 (p<0.01), PF4 (p<0.05)], and showed cytotoxic effects, as seen by increased LDH (p<0.001) and heme (p<0.0001) levels. We found that inflammation and endothelial cell activation were partly complement-dependent and inhibition of complement at the level of C3 by compstatin Cp40 significantly attenuated expression of ICAM-1 (p<0.01) and selectins (p<0.05). We show that complement activation plays an important role in the IONPs-induced thromboinflammatory response and that complement inhibition is promising in improving IONPs biocompatibility.

## Introduction

Iron oxide nanoparticles (IONPs) are used in several biomedical applications, including drug delivery, tumor therapy (1) and bioimaging (2). Their magnetic and biological properties make them suitable as contrast agents in magnetic resonance imaging (MRI) and as drug delivery agents (2). However, as for any systemically delivered particle, IONPs are recognized by the innate immune system as a potential threat to the host. The associated reactions can cause detrimental side effects or limit their therapeutic potential. Disturbed iron homeostasis, oxidative stress, cytotoxicity (3) and immunological responses (4) are potential adverse reactions to IONPs.

The innate immune response is a key factor in the *in vivo* performance of nanoparticles, as it is critical for the clearance of pathogens, damaged endogenous cells, and debris, or foreign particles introduced into the circulation (5, 6), with or without an associated inflammatory response (7). Upon contact with blood, a layer of proteins will instantly cover the nanoparticle’s surface, referred to as the protein corona (8). The protein corona, or “biological identity” of the nanoparticles, will depend on the synthetic identity, *i.e*., physical and chemical properties (9). The nanoparticles’ size, porosity, charge, and hydrophobicity will determine which proteins can adhere and their conformational state after binding. The identity of the protein corona will mediate further interactions with proteins, cells, and tissue (4). Typically, an overweight of proteins related to regulatory functions of the protein cascades in the blood, kept in their native conformation after binding, will promote biocompatibility (10, 11).

Innate immune recognition is served by pattern recognition molecules (PRMs), including the membrane-bound Toll-like receptors (TLRs) and soluble complement recognition molecules. The complement system is initiated *via* three different activation pathways (12). The classical and the lectin pathways are triggered by the binding of specific PRM, including C1q in the classical pathway and MBL, ficolin-1, -2, and -3, as well as collectin-10 and -11 in the lectin pathway. Activation progresses into the cleavage of the C4, C2, and C3. The alternative pathway lacks a specific PRM but is instead undergoing a continuous low-grade activation by hydrolysis of the internal thiol-ester, forming a C3(H_2_O) molecule mimicking C3b, forming a C3 convertase cleaving C3 into C3a and C3b. Furthermore, the C3 molecule has a unique property of distinguishing “self” from “non-self” surfaces by binding factor H to the former, inhibiting further activation, and factor B to the latter, promoting activation (13). All pathways converge into a common terminal pathway downstream of C3 with the cleavage of C5 and the formation of the terminal complement C5b-9 complex (TCC). Throughout the cascade, effector molecules are formed. The anaphylatoxins C3a and C5a activate cells *via* their associated receptors, C3aR, C5aR1, and C5aR2, respectively (12, 14). C3b acts as an opsonin in phagocytosis, and membrane-inserted C5b-9 has the potential to lyse sensitive cells or activate nucleated cells (15).

TLR- activation typically leads to NF-κB-activation and the cellular release of a variety of pro-inflammatory cytokines, including interleukin (IL)-1β, IL-6, and tumor necrosis factor (TNF) (2, 16). Likewise, complement activation, particularly C5a binding to C5aR1, is a potent pro-inflammatory signal for activating leukocytes and endothelial cells, leading to upregulation of activation-specific receptors and cytokine release. IL-8 is a chemokine often induced by C5a-C5aR1 activation (17).

Understanding the inflammatory response to nanoparticles, which can involve thrombus formation *via* the crosstalk to platelets and the coagulation cascade, is critical to avoid adverse reactions and improve biocompatibility. Here, we investigated the thromboinflammatory response mediated by IONPs in an *ex vivo* model involving human whole blood in interplay with endothelial cells and evaluated whether targeting key components in the inflammatory cascade could improve the IONPs’ biocompatibility.

## Materials and methods

### Reagents

IONPs suspensions of size 10, 20, and 30 nm were purchased from Ocean Nanotech, LLC (San Diego, CA). A stock combination mixture (1 mg/mL) of all three sizes was prepared in phosphate-buffered saline (PBS) containing Ca^2+^ and Mg^2+^ (Sigma Aldrich, Steinheim, Germany). Compstatin analog Cp40 [yI[CV(MeW)QDW-Sar-AHRC](NMe)I-NH2] was used to inhibit C3 cleavage (18) and PMX53 was used as a C5aR1 antagonist (19). Eritoran (E5564) was kindly provided by Eisai Co., Ltd. (Tokyo, Japan). The C5 inhibitor eculizumab (Soliris^®^) was obtained from Alexion Pharmaceuticals (Boston, MA). Anti-CD14 (r18D11), an IgG2/4 chimeric antibody, was produced in our laboratory as previously described (20). Complement inhibitors anti-C2 (mAb, clone 175-62) and anti–factor D (mAb, clone 166-32) were provided by Genentech (San Francisco, CA). Zymosan A from *Saccharomyces cerevisiae* was purchased from Sigma-Aldrich. Ultrapure lipopolysaccharide (LPS) from *Escherichia coli* strain 0111:B4 (smooth type) was obtained from InvivoGen (San Diego, CA).

### Whole blood sampling and plasma preparation

Human whole blood from healthy volunteers was collected in 4.5 mL cryotubes (Cryo Tube™ Vials; Thermo Scientific, Waltham, MA) containing lepirudin (50 mg/mL), a specific thrombin inhibitor (Refludan; Celgene, Uxbridge, UK), as an anticoagulant. Plasma was isolated by whole blood centrifugation at 3000 x g for 15 minutes, and a normal human plasma pool (NHP) was created by pooling plasma from six donors. All samples were stored at -80°C. To prepare platelet-rich plasma (PRP), whole blood was centrifuged at room temperature, 22°C at 180 x *g* for 10 minutes. PRP was immediately used in the respective applications.

### Human lung microvascular endothelial cells culturing

Human lung microvascular endothelial cells (HLMECs; Cell Applications Inc, San Diego, CA) of passage 4-6 were cultured in endothelial cell growth medium (Sigma-Aldrich) in T25 flasks. When the cells reached 80-90% confluence, they were washed and treated with 0.5 ml TrypLE™ (Thermo Fisher Scientific) for 7 minutes. Afterward, 200 000 or 300 000 HLMVEC cells/mL were seeded in 48-well Nunc plates (Thermo Fisher Scientific) pretreated with 0.1% gelatin solution (Sigma-Aldrich) for 5 minutes. The experiments were performed when the cells had reached confluence, typically after 48-72 hours.

### Complement activation by IONPs in human plasma

NHP (100 μL) was incubated with different-sized IONPs (10, 20, or 30 nm) in increasing concentrations (2, 20, and 200 μg/mL) or PBS. Zymosan (100 µg/mL) was used as a positive control. The samples were incubated for 30 minutes at 37°C in 1.8 mL cryotubes (Thermo Scientific). Ethylenediamine tetra-acetic acid (EDTA; 20 mM in final concentration) was added to prevent further activation. Finally, all samples were centrifuged at 3000 x *g* for 15 min at 4°C to remove the IONPs. In separate experiments, the same incubation conditions were used, however, a mixture of IONPs 10-30 nm was prepared and added in NHP at the following concentrations: 5, 10, 50, and 100 µg/mL. In this setup, we also included two inhibitors, Cp40 (20 μM) and eculizumab (100 μg/mL), in samples containing 100 µg/mL of IONPs. In another set of experiments, IONPs (10-30 nm, 100 μg/mL) or PBS were in separate experiments incubated in NHP with or without the anti-C2 mAb (72 μg/mL) and/or anti-factor D mAb (36 μg/mL), or 10 mM Mg^2+^/EGTA, or 10 mM EDTA for 30 minutes at 37°C. EDTA (20 mM final concentration) was added after incubation to prevent further activation. All samples were centrifuged at 3000 x *g* for 15 min at 4°C.

### Incubation of IONPs in human whole blood

Whole blood experiments were performed as previously described (21). The addition of 300 μL of blood in 1.8 mL cryotubes was followed by pre-incubation with each of the following inhibitors; Cp40 (20 μM), eculizumab (100 μg/mL), anti-CD14 (15 μg/mL), PMX53 (10 μg/mL) and eritoran (1 μM), or PBS for 5 minutes at 37°C. A mixture of IONPs 10-30 nm (100 µg/mL) or PBS was added, and the samples were incubated for four hours at 37°C. Zymosan (100 µg/mL) and LPS (10 ng/mL), in combination, were used as a positive control for complement activation and cellular activation. After incubation, the tubes were placed on ice, and EDTA (20 mM final concentration) was added. The samples were centrifuged at 3000 x *g* for 15 min, and plasma was immediately isolated and stored in aliquots.

### Incubation of IONPs on human lung microvascular endothelial cells in combination with the human whole blood model

At 70-90% confluence, the HLMECs were washed with tempered (37°C) PBS to remove cell debris and incubated with whole blood (150 µL) for four hours at 37°C with gentle shaking (50 rpm). For this procedure, whole blood was pre-incubated with the inhibitors described above, followed by the addition of the 10-30 IONPs mixture (100 µg/mL) or PBS. Before the addition, the IONPs were sonicated (UP200S Hielscher Ultrasonics, Teltow, Germany) for 8 minutes at 70% amplitude and 0.6 pulses. A combination of zymosan (100 µg/mL) and LPS (10 ng/mL) was used as a positive control. Separate wells were incubated in cell culture media (negative control) with and without TNF and IL-1β in combination (both at 10 ng/mL; Sigma-Aldrich) to evaluate endothelial cells’ activation. After incubation, the whole blood was removed, and EDTA (20 mM final concentration) was added. The plasma was isolated and stored at -80°C until analysis.

### Immunoassays

Complement activation markers TCC (soluble C5b-9) and C3bc were quantified by enzyme-linked immunosorbent assays (ELISA), as previously described in detail (22–24). Platelets’ soluble activation markers, platelet factor 4 (PF4), P-selectin (sCD62P), and neutrophil-activating peptide-2 (NAP-2) were measured by ELISAs purchased from R&D systems (Minneapolis, MN). Lactate dehydrogenase (LDH), a marker for cell toxicity, was analyzed by ELISA acquired from Abcam (Cambridge, UK). Cytokines analysis was performed using a 4-plex kit (R&D systems) for whole blood samples or a 27-plex kit (Bio-Rad, Hercules, CA) for whole blood/endothelial cells samples. Both kits included the following cytokines; Tissue Necrotic Factor (TNF), interleukin (IL)-1ß, IL-6, and IL-8, and were performed according to the instructions from the manufacturers. IL-8 was, additionally, measured by ELISA for whole blood samples (R&Ds systems).

### Flow cytometry

#### Monocytes and granulocytes

After 15 minutes of incubation of IONPs in whole blood on HLMVECs, 10 μL of whole blood were isolated and treated with EDTA (20 mM final concentration). Expression of the activation marker CD11b on monocytes and granulocytes was measured with a mouse anti-human CD11b PE (BD Biosciences; Franklin Lakes, NJ). The samples were stained with a mouse anti-human CD14 FITC (BD Biosciences) for gating the monocytes and a mouse anti-human CD45 PerCP (BD Biosciences) to exclude debris. All antibodies were incubated with the samples for 30 minutes. The erythrocytes were then lysed with AKC lysis buffer (155 mM NH_4_Cl, 10 mM KHCO_3_, 0.1 mM EDTA) in a ratio 1:20 for 15 minutes, and the leukocytes were fixed and resuspended with 0.5% (v/v) paraformaldehyde in PBS and 0.1% bovine serum albumin (BSA; Sigma-Aldrich).

#### HLMECs

After four hours of incubation, the HLMECs were washed with PBS twice and fixed with 0.5% paraformaldehyde. They were, afterward, washed with PBS and trypsinized with 20 µL trypsin-EDTA (Thermo Fisher Scientific). The cells were resuspended in 500 µL growth medium (2% fetal bovine serum) and centrifuged at 250 x *g* for 5 minutes. Before measurement, the cell pellet was resuspended in 100 µL in 0.1% BSA in PBS (PBSA). Endothelial cells’ activation was evaluated by measuring the expression of the activation markers P- and E-selectin and ICAM-1. The endothelial cells were stained for 30 minutes at 4°C in PBSA with a mouse anti-human MCAM PerCP (R&D Systems) for gating. A mouse anti-human ICAM-1 FITC (R&D Systems) and a mouse anti-human P/E-selectin PE (R&D Systems), which detects E-selectin and P-selectin on activated endothelial cells, were used for the detection of the activation markers.

#### Platelets

IONPs were added to PRP at a final concentration of 100 μg/mL and incubated for 15 minutes at 37°C. Platelets activated by TRAP-6 (25 μg/mL, Bachem, Bubendorf, Switzerland) were used as a positive control, while PBS addition served as a negative control. Additionally, we included Cp40, eculizumab, and PMX53 in the presence of IONPs (100 μg/mL). After incubation, EDTA (20 mM) and CTAD (8 mM trisodium citrate, 1.1 M theophylline, 26 mM adenosine, 14 mM dipyridamole; BD Biosciences) in combination were added to stop any further activation. C3c-containing fragments and the activation markers, P-selectin (CD62P) and CD63 were evaluated on the platelets using flow cytometry. The platelets were stained with mouse anti-human CD42a-PerCP (BD Biosciences) for gating. Mouse anti-human CD62P-PE (BD Biosciences) or mouse anti-human CD63-PE (BD Biosciences) were used for measuring platelet activation, and a polyclonal rabbit anti-human C3c-FITC (Dako) was used for measuring C3c-fragment deposition. The antibodies were incubated with the samples for 20 minutes and then fixed with 0.2% v/v paraformaldehyde in PBS containing 0.1% BSA.

All samples were run on an Acuuri C6 cytometer (BD Biosciences). The data were analyzed with Flow Jo, version 10 (Tree Star, Ashland, OR), and the results are given as the mean fluorescence intensity (MFI).

### Absorbance spectroscopy

Hemolysis was analyzed by recording the absorbance spectra in the 350 – 700 nm wavelength range using Nanodrop™ (Thermo Scientific). The data (λ = 414 nm) refer to the Soret peak of heme.

### Statistics

The data were analyzed using GraphPad (San Diego, CA) Prism version 9 for Mac by ordinary or repeated-measures one-way ANOVA with Dunnett multiple-comparison posttest for the comparison of multiple columns and paired t-tests for the comparison of two columns. A *p-value* < 0.05 was considered statistically significant.

### Ethics

The study was performed according to the ethical guidelines from the declaration of Helsinki. Sampling of human whole blood from healthy individuals for the preparation of plasma was approved by the ethical committee of the Norwegian Regional Health Authority, ethical permit REK#S-04114, 2010/934. Informed written consent was obtained from each donor.

## Results

### IONPs activated complement in human plasma in a dose-dependent manner

**Figure 1:**
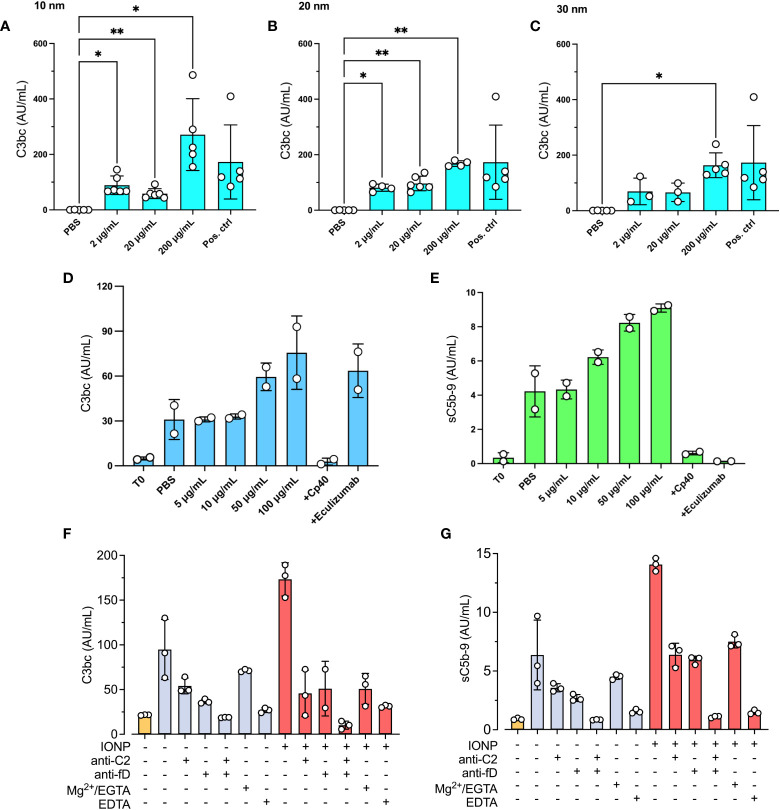
IONPs-induced complement activation in plasma. A pool of NHP was incubated with IONPs of various sizes; **(A)** 10 nm, **(B)** 20 nm, and **(C)** 30 nm in increasing concentrations; 2, 20, and 200 μg/mL. PBS was included as a negative control (PBS), and zymosan (100 μg/mL) as the positive control (Pos. ctrl). Complement activation was analyzed by measuring C3bc **(A–C)**. Separately, a mixture of IONPs containing all different sizes; 10, 20, and 30 nm (IONPs) in concentrations from 0-100 μg/mL, was incubated with NHP in the presence of Cp40 (20 μM) or eculizumab (100 μg/mL). One sample was immediately stopped by adding EDTA at the beginning of the incubation (T0) for comparison. PBS was included as a negative control (PBS). Complement activation was analyzed by measuring C3bc **(D)** and sC5b-9 **(E)** by ELISA after 30 minutes of incubation at 37°C. The values are shown as mean +/- standard deviation of n=3-6 **(A–C)** or n=2 **(D**, **E)**. *p < 0.05, **p < 0.01. **(F, G)** IONPs (10-30 nm, 100μg/mL, red bars) or PBS (blue bars) were incubated in NHP with or without anti-C2 mAb (72 μg/mL) and/or anti-factor D mAb (36 μg/mL), or 10 mM Mg^2+^/EGTA, or 10 mM EDTA for 30 minutes at 37°C. T0 was not incubated (yellow bar) and represents the starting value. Complement activation was evaluated by measuring C3bc **(F)** and sC5b-9 **(G)** by ELISA.

To evaluate whether IONPs promote complement activation, lepirudin-anticoagulated NHP was incubated with increasing concentrations of IONPs (2-200 μg/mL) of different sizes, 10, 20, or 30 nm, for 30 minutes, and C3bc plasma concentrations were measured. The highest concentration of IONPs, 200 μg/ml, led to significant complement activation (p<0.05-0.01) independently of the size of IONPs (**Figures 1A–C**). IONPs of 2 and 20 μg/mL caused significant complement activation (p<0.05-0.01) only when incubated with 10 nm- or 20 nm- sized IONPs (**Figures 1A, B**). Overall, the different sizes of IONPs activated complement to a similar degree and were in the following set of experiments combined in one mixture. In the next step, IONPs in increasing concentrations of 5-100 μg/mL were incubated with NHP. Two complement inhibitors were included, Cp40 and eculizumab, that block C3- and C5-cleavage, respectively, together with IONPs (100 μg/mL), and sC5b-9 formation was measured additionally to C3bc (**Figures 1D, E**). IONPs increased C3bc and sC5b-9 in a dose-dependent manner, and the most profound effect was observed for the highest concentration of IONPs (100 μg/mL) (**Figures 1D, E**). As expected, both inhibitors abolished sC5b-9 formation while Cp40, but not eculizumab, inhibited C3bc.

To investigate which complement activation pathway that is activated by IONPs, an anti-C2 antibody, inhibiting classical and lectin pathways, and an anti-factor D antibody, inhibiting the alternative pathway, were incubated with IONPs (100 μg/mL) for 30 minutes. Both antibodies inhibited activation to a similar degree (**Figures 1F, G**). After subtracting the levels of C3bc and sC5b-9 in non-incubated plasma (T0), C3bc was reduced by 84% and 81% by the anti-C2 and the anti-factor D respectively (**Figure 1F**). Similarly, sC5b-9 was decreased by 59% by the anti-C2 and by 61% by the anti-factor D (**Figure 1G**). Anti-C2 and anti-factor D in combination completely blocked C3- and terminal pathway activation. Furthermore, addition of 10 mM EGTA, which in the presence of 10 mM Mg^2+^ specifically blocks classical and lectin pathway activation by chelating Ca^2+^, reduced C3bc by 50% and sC5b-9 by 80%.

### Complement inhibitors effectively attenuated IONPs-induced inflammatory response in human whole blood

**Figure 2:**
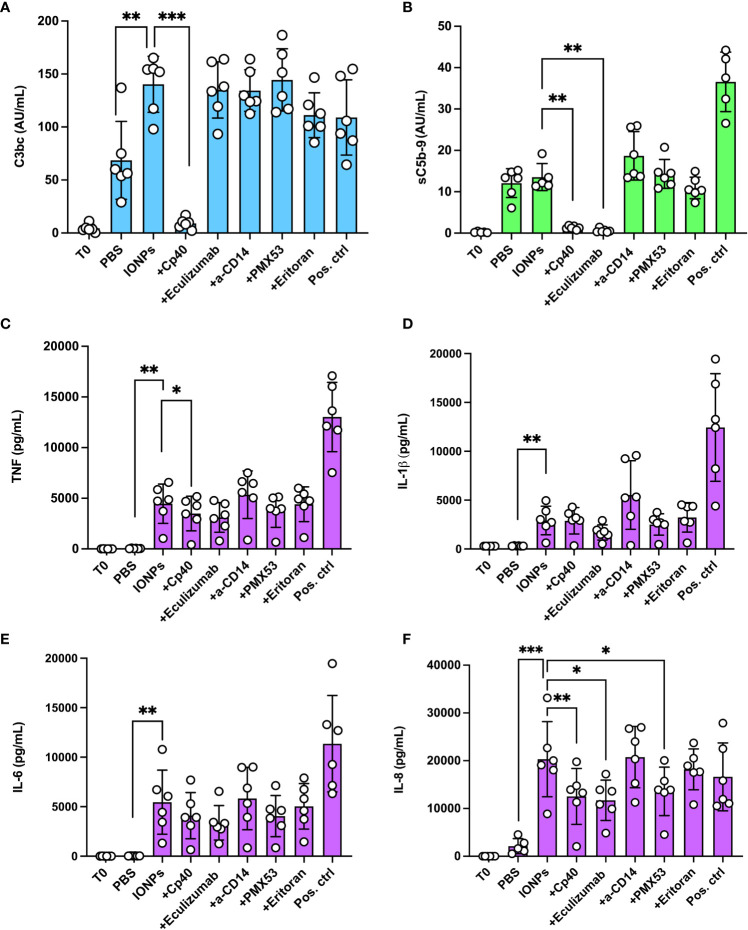
Effect of complement inhibition on complement activation and cytokine release by IONPs in whole blood. Human whole blood was incubated with a mixture of IONPs (100 μg/mL) of different sizes; 10, 20, and 30 nm in the presence of inhibitors; Cp40 (20 μM), eculizumab (100 μg/mL), a-CD14 (15 μg/mL), PMX53 (10 μg/mL) or eritoran (1 μM) for four hours at 37 °C. Zymosan (100 μg/mL) and LPS (10 ng/mL) in combination served as a positive control (Pos. ctrl) and PBS as a negative control (PBS). One sample was immediately stopped by adding EDTA at the beginning of the incubation (T0) for comparison. Complement activation was evaluated by measuring the formation of C3bc **(A)** and sC5b-9 **(B)** by ELISA. **(C)** TNF, **(D)** IL-1β and **(E)** IL-6 were evaluated by 4-plex Luminex® assay and **(F)** IL-8 was measured by ELISA after four hours of incubation at 37°C The values are shown as mean +/- standard deviation of n=6. *p<0.05, **p < 0.01, ***p < 0.001.

Human whole blood was incubated with IONPs (100 μg/mL) of combined sizes (10, 20, and 30 nm) for four hours. IONPs significantly increased C3bc levels (p<0.01) by 105% (**Figure 2A**), however, they did not induce a significant increase in sC5b-9 formation (**Figure 2B**). Cp40 resulted in a significant decrease of both C3bc (p<0.001) and sC5b-9 (p<0.01), while eculizumab resulted in a significant reduction of sC5b-9 formation (p<0.01). Anti-CD14, eritoran, and PMX53 did not significantly influence the level of C3bc or sC5b-9.

IONPs led to a robust release of pro-inflammatory cytokines; TNF (p<0.01), IL-1β (p<0.01), IL-6 (p<0.01), and IL-8 (p<0.001) were all significantly increased by IONPs (**Figures 2C–F**). Inhibition with Cp40, eculizumab, and PMX53 significantly reduced IL-8 (**Figure 2C**) by 38% (p<0.01), 42% (p<0.05), and 33% (p<0.05), respectively. Inhibition with Cp40 significantly decreased TNF (**Figure 2D**) production by 22% (p<0.05). Eculizumab lowered TNF levels, but this reduction was not statistically significant (p=0.059). The release of IL-1β (**Figure 2E**) and IL-6 (**Figure 2F**) was also lower in all three complement inhibitor samples, but none of these were statistically significant. Blockade of TLR-signaling with a-CD14 or eritoran did not significantly influence the concentration of any of the analyzed cytokines.

### Complement inhibitors reduced IONPs-induced inflammatory response in human whole blood with endothelial cells

**Figure 3:**
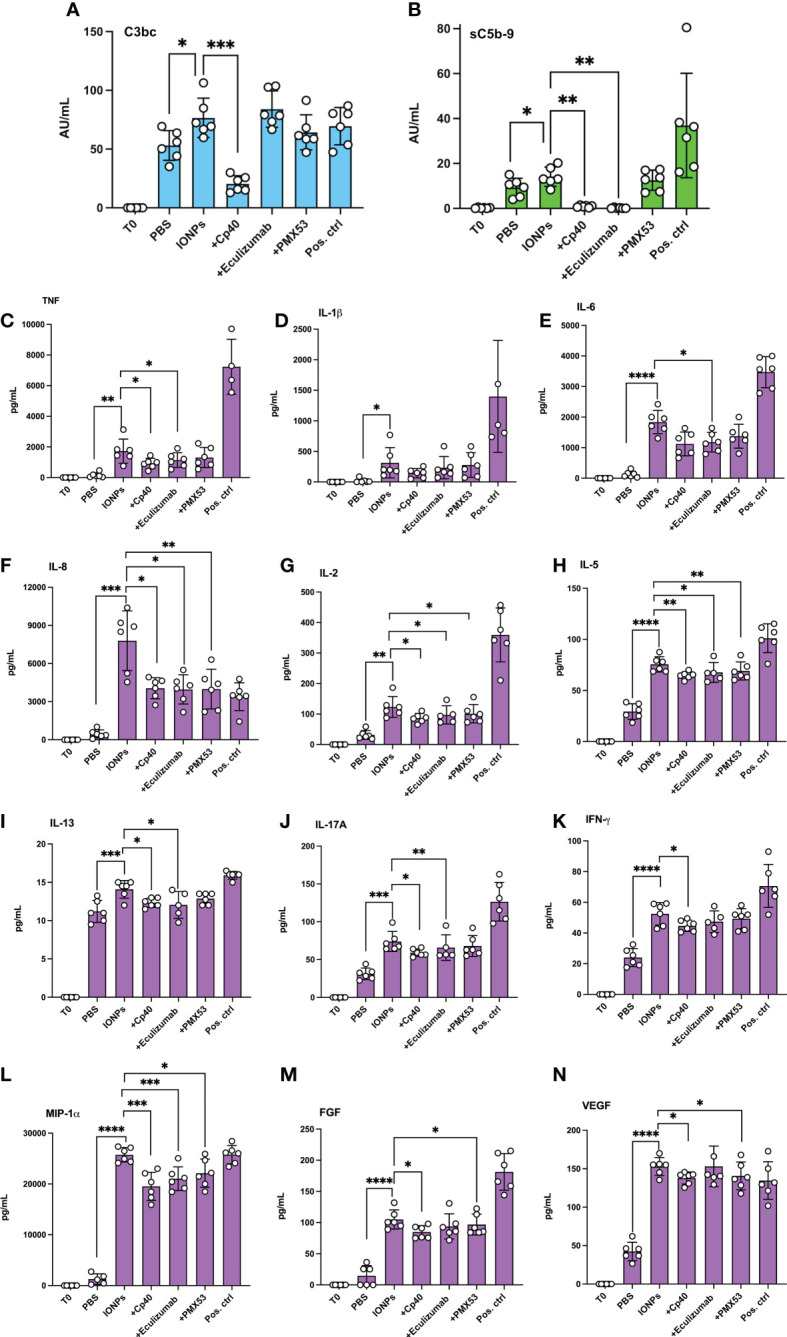
IONPs-induced complement activation and cytokine release in whole blood interplay with endothelial cells. Human whole blood was incubated together with HLMECs and a mixture of IONPs (100 μg/mL) of different sizes; 10, 20, and 30 nm in the presence of inhibitors; Cp40 (20 μM), eculizumab (100 μg/mL) or PMX53 (10 μg/mL) for four hours at 37 °C. Zymosan (100 μg/mL) and LPS (10 ng/mL) in combination served as a positive control (pos. ctrl) and PBS as a negative control (PBS). One sample was immediately stopped by adding EDTA at the beginning of the incubation (T0) for comparison. Complement activation was evaluated by measuring the formation of C3bc **(A)** and sC5b-9 **(B)** by ELISA. Plasma was analyzed with a 27-plex cytokine Luminex® assay, and the concentration of TNF **(C)**, IL-1β **(D)**, IL-6 **(E)**, IL-8 **(F)**, IL-2 **(G)**, IL-5 **(H)**, IL-13 **(I)**, IL-17A **(J)**, IFN-γ **(K)**, MIP-1α **(L)**, FGF **(M)** and VEGF **(N)** are presented. The values are shown as mean +/- standard deviation of n=6. *p<0.05, **p<0.01, ***p<0.001, ****p<0.0001.

We further studied the inflammatory response induced by IONPs in the human whole blood model, but this time in combination with HLMECs to include the whole blood and endothelial cells interaction. After four hours of incubation, IONPs-induced complement activation, as measured by C3bc (**Figure 3A**) and sC5b-9 (**Figure 3B**), was significantly elevated (p<0.05). Inhibition with Cp40 significantly attenuated both C3bc (p<0.001) and sC5b-9 levels (p<0.01). Eculizumab completely blocked sC5b-9 formation (p<0.01), while PMX53 showed no effect, as expected.

Cytokine release was induced by IONPs in human whole blood accompanied by endothelial cells, as demonstrated by a significant increase in the production of most cytokines, including TNF, IL-1β, IL-6, IL-8, and IL-2, as well as production of IFN-γ. Overall, 21 out of the 27 analyzed markers were significantly increased due to the presence of IONPs (**Figures 3C–N**; **Supplementary Figure 1**). Interestingly, IP-10 and eotaxin were significantly reduced by the IONPs (**Supplementary Figure 1**). Cp40, eculizumab, and PMX53 significantly attenuated the release of IL-8, IL-2, IL-5, and MIP-1α (**Figure 3**). In addition, Cp40 and eculizumab significantly also reduced TNF, IL-17A, IL-13 and IL-12, Cp40 alone reduced IFN-γ, VEGF and FGF formation while eculizumab alone significantly decreased IL-6, as well as GM-CSF and MCP-1 (**Figure 3**; **Supplementary Figure 1**). Additionally, PMX53 showed a significant attenuating effect on FGF, VEGF, and GM-CSF (**Figure 3**; **Supplementary Figure 1**). No inhibitory effects were observed on IL-1β and IL-1Ra (**Figure 3**; **Supplementary Figure 1**).

### IONPs-induced activation of endothelial cells and monocytes was complement-dependent

**Figure 4:**
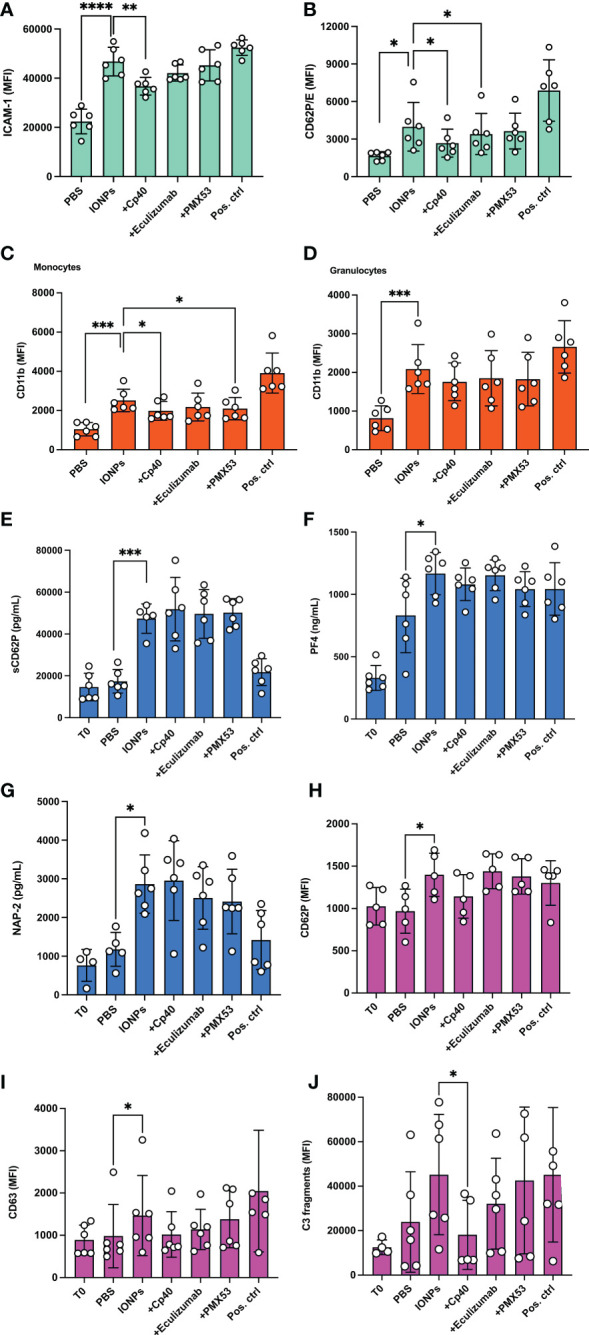
IONPs-induced endothelial cell-, monocyte-, granulocyte- and platelet activation. Human whole blood was incubated together with HLMECs and a mixture of IONPs (100 μg/mL) of different sizes; 10, 20, and 30 nm in the presence of inhibitors; Cp40 (20 μM), eculizumab (100 μg/mL) or PMX53 (10 μg/mL) for four hours at 37°C. Zymosan (100 μg/mL) and LPS (10 ng/mL) served as a positive control (Pos. ctrl) for endothelial cells and monocytes, respectively. PBS was used as a negative control (PBS). Endothelial cell activation was evaluated by measuring ICAM-1 **(A)** and CD62P/E **(B)** expression by flow cytometry. Cell surface activation marker CD11b was measured on monocytes **(C)** and granulocytes **(D)** by flow cytometry. Plasma was analyzed by ELISA for soluble markers of platelet activation; sCD62P **(E)**, PF4 **(F)**, and NAP-2 **(G)**. Additionally, PRP was isolated from the same donors and incubated separately under the same experimental conditions for 15 minutes. TRAP-6 (25 μg/mL) was used as a positive control (Pos. ctrl) and PBS as a negative control (PBS). Cell surface activation markers CD62P **(H)** and CD63 **(I)** and C3c fragment deposition **(J)** were measured on platelets by flow cytometry. The values are shown as mean +/- standard deviation of n=6. *p<0.05, **p<0.01,***p < 0.001, ****p<0.0001.

To evaluate the effect of IONPs on endothelial cells’ activation, ICAM-1 and CD62P/E were measured by flow cytometry after four hours of incubation in the presence of IONPs. Expression of both ICAM-1 (**Figure 4A**) and CD62P/E (**Figure 4B**) was significantly elevated (p<0.0.0001 and p<0.05, respectively). Inhibition by Cp40 significantly attenuated ICAM-1 by 21% (p<0.01) and CD62P/E by 33% (p<0.05), respectively. Eculizumab significantly reduced only CD62P/E expression (p<0.05), while PMX53 did not influence endothelial cell activation. Monocytes and granulocytes were isolated from the whole blood after the first 15 minutes of incubation, and the cell surface activation marker CD11b was evaluated by flow cytometry. IONPs induced a substantial CD11b increase (p<0.001) on both monocytes and granulocytes. Monocyte activation was significantly reduced by Cp40 (p<0.05) and PMX53 (p<0.05), but not eculizumab (p=0.051) (**Figure 4C**). Complement inhibition did not significantly attenuate granulocyte activation (**Figure 4D**).

### Complement inhibitors did not attenuate IONPs-induced activation of platelets

Platelets play an important role in the thromboinflammatory response. Therefore, we evaluated how IONPs affect their activation in whole blood. Soluble platelet activation markers, sCD62P, PF4, and NAP-2, were analyzed in plasma isolated from the whole blood after incubation on endothelial cells for four hours. IONPs significantly increased all three markers (p<0.05-0.001), and no inhibitory effect was observed due to complement inhibition, as measured by C5-, C3-, or C5aR1-blockade (**Figures 4E–G**). In a separate set of experiments, PRP was incubated for 15 minutes with IONPs. Platelet activation, evaluated by measuring surface activation markers CD62P and CD63 by flow cytometry, was increased by IONPs (p<0.05), but it was not significantly reduced by the presence of the complement inhibitors (**Figures 4H, I**). Finally, we measured platelet C3-fragment deposition and found increased values in the presence of IONPs, however, the difference was not statistically significant (**Figure 4J**), most likely due to the biological variation amongst donors, since this increase was significantly (p<0.05) reduced by the C3 inhibitor Cp40,

### IONPs caused cytotoxic injury

**Figure 5:**
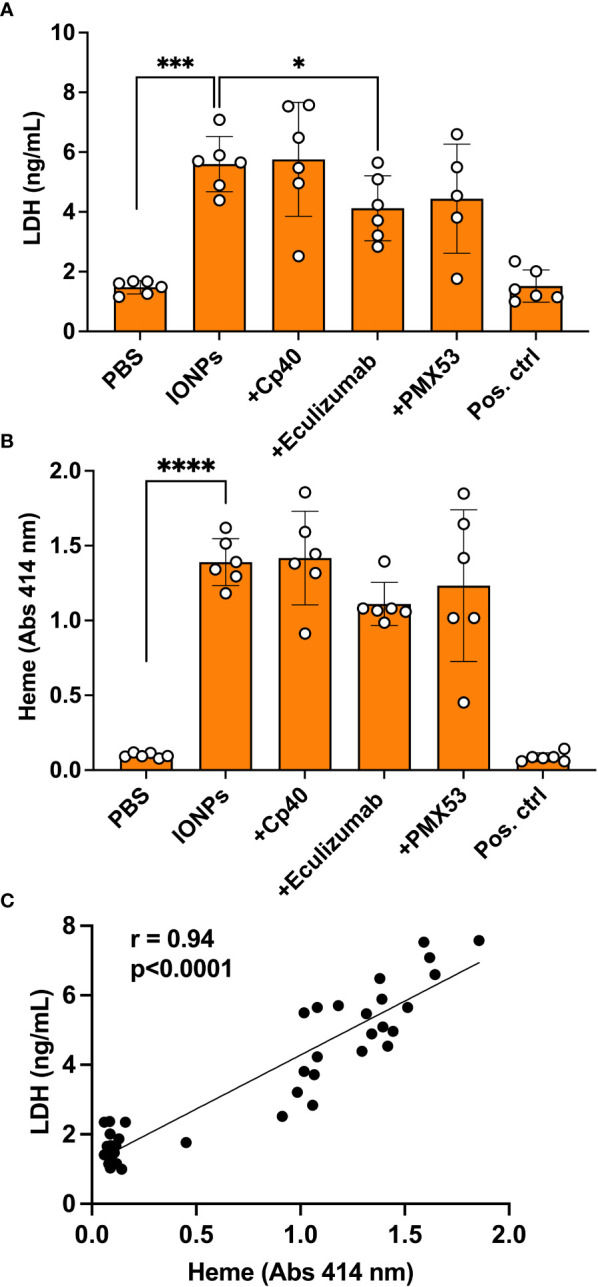
IONPs-induced cytotoxic effect. Human whole blood was incubated together with HLMECs and a mixture of IONPs (100 μg/mL) of different sizes; 10, 20, and 30 nm in the presence of inhibitors; Cp40 (20 μM), eculizumab (100 μg/mL) or PMX53 (10 μg/mL) for four hours at 37 °C. Zymosan (100 μg/mL) and LPS (10 ng/mL) in combination served as a positive control (Pos. ctrl) and PBS as a negative control (PBS). Cytotoxicity was evaluated in plasma by measuring LDH concentration by ELISA **(A)** and heme **(B)** by absorbance spectroscopy at 414 nm. The correlation between LDH and heme is shown **(C)**. The values are presented as mean +/- standard deviation of n=6. *p<0.05, ***p<0.001, **** p<0.0001.

Since we visually observed increased hemolysis in the samples containing IONPs after incubation in whole blood on endothelial cells, we decided to measure LDH as a marker of cell damage and the total amount of heme as a marker of hemolysis. IONPs significantly elevated LDH release (p<0.001) (**Figure 5A**) and heme concentration (**Figure 5B**) (p<0.0001) in comparison to the control. A positive correlation (r = 0.94) was found between LDH and heme levels (**Figure 5C**). Eculizumab significantly decreased the LDH level (**Figure 5A**) (p<0.05). No significant differences were detected for any of the other inhibitors.

## Discussion

The innate immune system interactions with nanoparticles are of essential importance in current basic and clinical science. Thus, in the present study, we aimed to elucidate critically important mechanisms of this interaction that have not previously been approached under close to physiological conditions. We evaluated the thromboinflammatory response by IONPs using a human whole blood model in the presence of endothelial cells. We found that IONPs resulted in a substantial increase in the inflammatory reaction, including complement activation cytokine release and activation of endothelial cells, monocytes, granulocytes, and platelets, and led to increased LDH as a marker of cytotoxicity. We also demonstrated an important attenuating effect on the inflammatory response by complement inhibition with the C3 inhibitor Cp40 and the C5aR1 inhibitor PMX53.

Initially, we tested whether the IONPs-induced complement activation was dose and size-dependent since the physical and chemical properties, e.g., size, of NPs can influence the formation of the protein corona and, thus, the subsequent inflammatory response (25). For that purpose, we incubated NHP with increasing concentrations of IONPs of various sizes; 10, 20, or 30 nm. The strongest activation of complement was observed for the 10 nm-sized particles and the lowest for the 30 nm-sized particles in a dose-dependent manner. This can most likely be explained by the higher surface area per weight for the smaller particles compared to the larger ones, which for the 10 nm particles is three times larger compared to the 30 nm particles for the same weight. Though, smaller particles have a lower ability to bind the large C1q and MBL recognition molecules of the CP and LP (26), this might be a compensatory factor for the larger particles. Therefore, in the following experiments, we prepared a mixture of IONPs containing different sizes to eliminate any size-dependent effect. By specifically blocking the classical- and lectin pathways by a C2-antibody, and the alternative pathway by a factor D-antibody, we found that both the classical/lectin pathways and the alternative pathway could be triggered independently from each other by the IONPs, and contribute to both C3- and terminal pathway activation.

For all particles, we saw a strong dose-dependent response on complement activation with the maximum response for IONPs in a concentration of 200 μg/mL. This is in contrast to a previous study, which reported that 10 nm-sized IONPs induced the strongest activation of complement, including sC5b-9 and C3a, at 10 µg/mL, while no clear activation was seen at 100 µg/mL (27). Noteworthy, sC5b-9 levels in our experiments were generally lower than C3bc levels, and IONPs, when incubated alone with whole blood, did not lead to a significant increase in sC5b-9. Considering the surface deposition of C3b as a promoter for the complement convertases to switch substrate specificity from C3 to C5 (28), the limited capacity of small particles to offer a platform for C3b-deposition (26), similar to non-solid surfaces (29), could explain the more efficient C3- than C5-activation on IONPs.

IONPs induced a strong inflammatory response in whole blood. Expression of pro-inflammatory cytokines, IL-8, TNF, IL-1β, and IL-6 were increased in whole blood, similar to what was reported previously (27). The C3 inhibitor compstatin analog Cp40 significantly decreased the production of IL-8 and TNF but did not show a similar effect on IL-1β, and IL-6. Notably, inhibition of C5 and C5aR1 reduced the levels of IL-8. C3-inhibition blocks the generation of all effector mechanisms from C3 and downstream, i.e., C3a, C3b, iC3b, C3c, and C3d, as well as C5a and MAC, whereas C5-inhibition blocks C5a and MAC, and C5aR1-blockade will specifically block C5a-dependent activation of the C5aR1. Thus, C3 inhibition will also block downstream C5 activation. Blocking of TLR4/MD2 or CD14 did not affect cytokine concentrations. Therefore, the cytokine secretion induced by IONPs was not TLR-mediated, consistent with previous findings (16).

The cytokine release was also evaluated in samples incubated in whole blood with endothelial cells. Overall, the majority of the cytokines measured were significantly elevated. On the contrary, two chemokines, IP-10 and eotaxin were significantly reduced. Whether this reduction was a biological response or dependent interference with the antibody-based detection system remains unsolved. Others have also observed a significant increase of IL-1β, TNF, IL-6, MCP-1, IL-8, and MIP-1β in the presence of IONPs, but they did not detect an increase in other cytokines (27). However, those experiments were performed in whole blood, whereas we used whole blood exposed to endothelial cells, which could contribute to the cytokine release. Inhibition of C3, C5, and to a lesser degree, C5aR1 attenuated, but not completely inhibited, a broad range of cytokines.

Furthermore, ICAM-1 and P/E-selectin, adhesion molecules upregulated on endothelial cells upon activation, were attenuated by C3-inhibition. C5-inhibition could only reduce P/E-selectin upregulation, while C5aR1 inhibitor did not show a significant inhibitory effect. ICAM-1 and E-selectin are expressed in response to several cytokines, such as TNF and IL-1β (30, 31). In our model, cytokine release was mainly complement-mediated. These findings support that C3 plays an important role in endothelial cell activation. Endothelial cell activation might also be potentiated directly from local complement deposition and sublytic MAC-formation on stressed endothelial cells (32). It is not clear from our data whether the IONPs also would trigger complement deposition on the endothelial cells. However, other factors, like cell-free heme, have been shown to prime primary endothelial cells for C3- and C5b-9 deposition (33, 34). Inhibition of C3 and C5aR1 attenuated upregulation of the cell-surface molecule CD11b on monocytes, further highlighting the anti-inflammatory potential of targeting C3 and C5aR1.

While IONPs have been thoroughly studied, there are still open questions regarding how they influence platelet function (35). We measured soluble and surface platelet activation markers. All markers were significantly increased in the presence of IONPs, however, complement inhibition did not show a significant effect on platelet activation. Platelet aggregation caused by metallic NPs, including IONPs, has been shown before (36). Studies, though, have observed that metal NPs can inhibit (37) or not affect all platelet functions (38). Nevertheless, these contradicting observations can be due to the physiological state of platelets prior to exposure to NPs (39). Our blood model, when combined with endothelial cells, is as close to physiologic conditions as it is possible to obtain in an *ex vivo* system.

Excess iron can produce reactive oxygen species (ROS) *via* Fenton and Haber-Weiss reactions (40, 41). Oxidative stress can lead to intracellular and DNA damage (2). Eventually, cell death will take place through a process called ferroptosis (42). IONPs can mediate ROS generation either at the particle surface or by the intracellular release of Fe^2+^/Fe^3+^ ions (2). Oxidative stress has been identified as the primary contributor to IONPs-mediated cytotoxicity (2). Therefore, IONPs were assessed by evaluating LDH in the present study, a marker of cell integrity, and heme, a byproduct of hemolysis. Both markers were significantly increased in parallel in the presence of IONPs. Their strong positive correlation indicates that LDH is derived primarily from red blood cells. Interestingly, the inhibition of C5 attenuated LDH and heme levels. Similar cytotoxic effects have been demonstrated earlier for IONPs, however, these effects can be counteracted by modulating surface coating and particle size (43).

In conclusion, IONPs induced a thromboinflammatory response in whole blood in the presence of endothelial cells, partly in a complement-dependent manner. Inhibition of primarily C3, but also C5 and C5aR1 could attenuate this response, but other mechanisms may add to this response, e.g., since the platelet activation seemed to be complement-independent (44). CD14 and TLR4 did not mediate this response since blocking these targets showed no inhibitory effect. Inhibition of C3 attenuated the activation of endothelial cells and monocytes most effectively, while complement inhibition did not reduce platelet activation. Finally, C5 inhibition could decrease IONP cytotoxic effects. Altogether, our findings show that complement inhibition is a promising tool for limiting the inflammatory reactions of IONPs.

Furthermore, there is a need to develop *in vitro* assays with good *in vivo* predictability (45). Our model allows us to study more complex responses compared to the *ex vivo* whole blood model, including the crosstalk between innate immunity and endothelial cells. However, it lacks several *in vivo* vascular characteristics, such as blood flow.

## Data availability statement

The raw data supporting the conclusions of this article will be made available by the authors, without undue reservation.

## Ethics statement

The studies involving human participants were reviewed and approved by Norwegian Regional Health Authority. The patients/participants provided their written informed consent to participate in this study.

## Author contributions

AG and MB designed and performed experiments and wrote the paper. CM and DS performed or/and designed or/and supervised experiments. TW and JL provided study material and edited the manuscript. TM provided critical discussions and edited the manuscript. PN designed and supervised the project, wrote the paper. All authors contributed to the article and approved the submitted version.
