# Supplementary material for: In vitro evaluation of iron oxide nanoparticle-induced thromboinflammatory response using a combined human whole blood and endothelial cell model
Source: Front Immunol. 2023 Apr 4;14:1101387. doi: 10.3389/fimmu.2023.1101387 (PMC10111002; doi:10.3389/fimmu.2023.1101387)
Supplement: Supplementary file 1 [file Image_1.pdf]

Supplementary Material

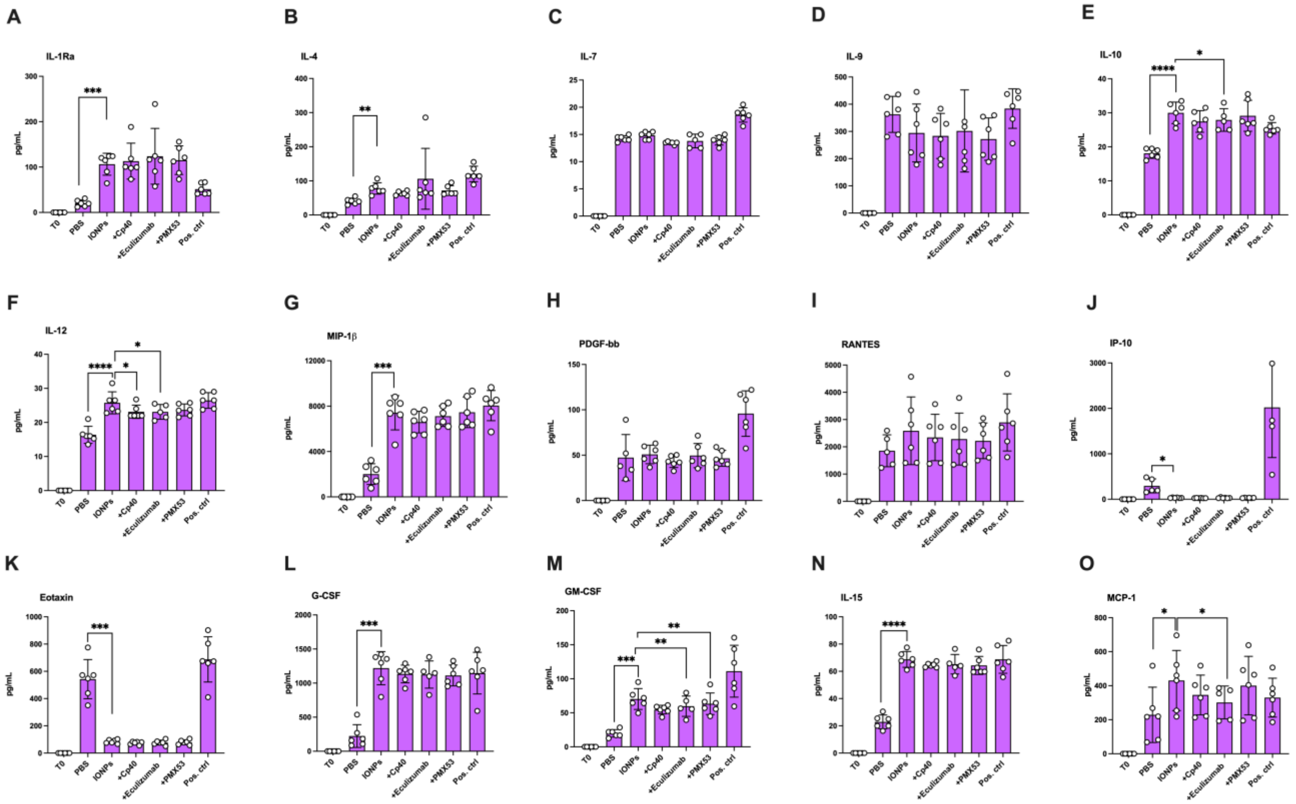

Supplementary Figure 1. IONPs-induced cytokine release in whole blood in interplay with endothelial cells. Human whole blood was incubated together with HLMECs and a mixture of IONPs (100 µg/mL) of different sizes; 10, 20, and 30 nm in the presence of inhibitors; Cp40 (20 µM), eculizumab (100 µg/mL) or PMX53 (10 µg/mL) for four hours at 37℃. Zymosan (100 µg/ml) and LPS (10 ng/ml) in combination served as a positive control (Pos. ctrl) and PBS as a negative control (PBS). One sample was immediately stopped by adding EDTA at the beginning of the incubation (T0) for comparison. Plasma was analyzed by a 27-plex cytokine Luminex® assay, and the concentration of IL-1Ra (A), IL-4 (B), IL-7 (C), IL-9 (D), IL-10 (E), IL-12 (F), MIP-1β (G), PDGF-bb (H), RANTES (I), IP-10 (J), Eotaxin (K), G-CSF (L), GM-CSF (M), IL-15 (N) and MCP-1 (O) are presented. The values are shown as mean +/- standard deviation of n=6. *p<0.05, **p<0.01, ***p<0.001, ****p<0.0001.
